# A Blockchain Based Secure IoT System Using Device Identity Management

**DOI:** 10.3390/s22197535

**Published:** 2022-10-04

**Authors:** Fariza Sabrina, Nan Li, Shaleeza Sohail

**Affiliations:** 1 School of Engineering and Technology, Central Queensland University, Sydney, NSW 2000, Australia; 2 School of Information and Physical Sciences, The University of Newcastle, Callaghan, NSW 2308, Australia; 3 King’s Own Institute, Sydney, NSW 2000, Australia

**Keywords:** IoT, authentication, identification, fuzzy extractor, IoT security, blockchain

## Abstract

Sharing data securely and efficiently has been identified as an issue in IoT-based smart systems such as smart cities, smart agriculture, smart health, etc. A large number of IoT devices are used in these smart systems and they produce a large amount of data. IoT devices generally have limited storage and processing capabilities, and configuring any security techniques on these devices is a challenge. In this paper, we propose a novel device identity management approach for blockchain-based IoT systems that provides data security in two ways. Firstly, a lightweight time-based identification protocol that uses hub identification for validating data. Secondly, data storage is augmented with an effective blockchain application for providing easy access and immutability for data sharing among multiple parties. Our initial prototype implementation shows that: our identity management approach can be implemented in large scale settings, our system can be effectively implemented in blockchain platforms, and our performance evaluation result shows that the prototype fulfills system requirements adequately.

## 1. Introduction

Internet of Things (IoT)s are becoming an essential part of everything around us as an innovation to put forward varieties of smart systems to improve quality of life. The use of smart agriculture, smart home and smart city systems have provided huge benefits in terms of convenience, real time environmental monitoring, dynamic fault prevention and many more. By using IoT, smart systems have changed industry requirements by providing the means for automating data collection, data sharing, monitoring and response to changed conditions.

Over time, with increased use, IoT devices have evolved in terms of communication, processing power and integration with other systems. Still, most IoT devices are hugely resource constrained when it comes to battery life, processing power and storage capability. Due to this, most IoT devices lack advanced mechanisms for security and privacy, and hence can be the weakest link when it comes to the overall system security by being vulnerable to attacks from cyber-criminals and hackers [1]. A considerably explored area of research for securing IoT devices is the use of machine learning to detect security issues [2,3]. Most machine learning approaches learn device and traffic features from existing data for detecting security attacks using classification methods [4,5]. Moreover, IoT sensors can provide data that can be effectively used for making real time decisions. However, when it comes to data sharing, most of these IoT based systems rely on centralized repositories that have a number of vulnerabilities that make data insecure especially in multi-party environments [6].

Machine learning-based classification groups devices and their data after learning the distinguishing features from existing datasets without uniquely identifying a device. Device identification is a process that needs to verify that a particular device is sending data so a receiver can be sure that the data is sent by the right source. Such identification is essential for ensuring data authenticity and eliminating risks due to rogue devices (unauthorized devices claiming falsified identity or authorized devices that have been compromised) [7].

In our work, we propose a new IoT device identification protocol based on fuzzy extractors using the timing information. The protocol can identify an IoT device with overwhelming accuracy and it can provide strong evidence of message authentication. When compared with the existing approaches (e.g., [7]), the proposed protocol generates a message authentication code with negligible overhead. For the purpose of effective data sharing, while establishing full trust among all the parties, blockchain and smart contracts have been proposed for the IoT systems. Immutability, availability and transparency in data sharing are some of the blockchain features that make it most suitable for this purpose.

We are proposing a fuzzy extractor and blockchain-based system that ensures data security in two aspects: firstly, by using an identification protocol based on timing information and secondly, by using blockchain for storing data information immutably. The proposed identification protocol allows the user to register the hub with the controller by sharing a secret key. This step does not require storing of any key information on the hub, which relaxes the device capability requirements. Due to the fact that sensors and hubs are lightweight devices, the protocol introduces minimum overhead in terms of storage and processing. Using the timing information, the controller can identify each hub individually and authenticate the data sent by that hub. After authenticating the data, the controller stores data information in blockchain to provide not only data integrity, but also improved data accessibility.

The main contributions of this paper are:

- We propose a novel framework for ensuring data and device security of an IoT system using blockchain and a fuzzy extractor.
- We propose and provide a detailed analysis of the lightweight time-based identification protocol (LiTBIP) for securing small IoT devices.
- We created a Raspberry Pi-based prototype of the proposed system and an Ethereum-based blockchain application. We provided a performance analysis of the LiTBIP protocol and blockchain application of the proposed framework.

The rest of this paper is organized as follows. Section 2 briefly reviews the related work of the proposed system. Section 3 provides an overview of the proposed system and describes the system modules. Section 4 gives some formal definitions and the notations of the proposed protocols. In Section 5, we describe the proposed system with the underlying protocols, and we demonstrate the implementation of our system in Section 6. Section 7 shows the performance evaluation of the proposed system, and Section 8 concludes this paper and presents the future work.

## 2. Related Work

In this section, we briefly discuss related research in the area of IoT device classification and identification. In the end, we will briefly discuss a few related decentralized user identification systems.

Behavioral fingerprinting approach for IoT device identification is proposed based on machine learning methods [2]. The authors used network traffic behavior/activities of IoT devices as features to train the machine learning model. The goal is to identify devices with similar functionality. Packet header features and payload based features were used as network features to classify the IoT devices and k-nearest-neighbors, decision trees, gradient boosting and majority voting methods were used for device type classification. Performance evaluation shows that the proposed method achieves high accuracy in fingerprinting IoT device types. A Convolution Neural Networks Long Short-Term Memory Network (LSTM-CNN) based approach is proposed that uses discriminating features from network traffic flows for classifying IoT devices with promising results [3].

Four different tree-based and neural network-based machine learning models (such as Random Forest (RF), 2D CNN, Decision Tree and Fully connected Neural Network) were compared for identifying IoT devices using network behavior [4]. The finding from the experimental work emphasizes the need of updating the models continuously, as the accuracy degrades over time when the model is tested on data that is outside the training set. Yousefnezhad et al. [5] proposed a framework that uses packet header information, sensor measurement and statistical feature sets as features for classifying and identifying IoT devices. Several machine learning methods such as RF, Support Vector Machine (SVM), and Logistic Regression (LR) were used for training the model. The experimental results show that the accuracy is better for the measurement-header model.

Several approaches have been proposed for specific device identification using feature-based statistical learning [7]. Different features among the Radiometric Fingerprint (RF) have been used for this purpose. A phase locking mechanism is proposed to observe the control voltage of the oscillator and the steady state control voltage value is used as RF for device identification [8]. RF-DNA (Distinctive Native Attributive) method assumes that the statistics of consecutive sub-regions in received signals from a wireless device may provide device identification and a number of different approaches have been proposed based on the general idea of RF-DNA [9,10]. The feature-based statistical learning methods for device identification has a number of open issues. Some such issues are: influence of obstacle movement in propagation path on channel state features, (a) manual effort required to extract features and high order statistics, (b) no guarantee of features being time invariant, (c) limited large scale datasets for training and testing ML approaches and so on [7].

Marchal et al. [11] proposed a system named AuDI for fingerprinting device types in an IoT system. In the proposed system, no prior information is required, and information from periodic communication traffic was used for device identification using an unsupervised machine learning method. The experimental result shows that the proposed system can identify devices with 98.2% accuracy.

Hamad et al. [12] proposed a passive device fingerprinting technique for IoT systems. In the proposed system, the fingerprint is created from features selected using both packet headers and payload information. A supervised machine learning method is used for detecting behavioral changes in devices and hence identifying a rogue device for further monitoring. The proposed technique can also identify devices from the same model and vendor with 90.3% accuracy.

Yin et al. [13] proposed IoT ETEI, a deep learning-based automatic end-to-end IoT device identification method. The proposed method is based on CNN+BiLSTM and uses spatial and temporal features extracted from traffic to identify devices. The author argues that since the proposed method does not require any prior knowledge for feature engineering, it is efficient in terms of low overhead. The performance of the proposed model was evaluated using two publicly available data sets (UNSW smart home traffic dataset containing 22 IoT devices and YourThings smart home traffic dataset containing 17 IoT devices) and the results show that the proposed method achieves accuracy rates of 99.91% and 99.68%, respectively.

Miettinen et al. [14] proposed a system named IoT SENTINEL to identify the types of IP based IoT devices being connected to a network. In this work, the device type is defined as a combination of device model and software version, and device fingerprinting was based on passive observation of network traffic. Twenty-three packet features were used for feature engineering, and all of these features were extracted from encrypted traffic (which does not require to rely on packet payload). The author argues that the proposed system has low overhead and can identify devices effectively.

Gong et al. [15] proposed a blockchain-based identity authentication framework for IoT devices. In the proposed system blockchain is used to store device identity information, and a Blockchain of Things (BCoT) Gateway was proposed for recording authentication transactions. This work uses device traffic flow for the device recognition model. The performance of the proposed model was evaluated using a public dataset and the results show that the proposed system can recognize devices with an accuracy rate of over 95%.

For device management, blockchain is used to identify and register IoT devices in a smart grid [16]. Multiple consensus algorithms are also studied and compared when employed in the proposed system. Numerous machine learning approaches can be used by hackers to deanonymize users based on the transaction submitted by users to blockchain. In order to anonymize user identification based on the transaction history in blockchain-based IoT applications, an obfuscation-based technique is proposed. As IoT devices generate transactions based on a time pattern so different timestamp related obfuscation methods are used to break the pattern and results show a significant reduction in informed and blind attacks [17].

A distributed authentication system using blockchain is proposed that provides a means to login to any application that supports that authentication system. A smart contract in the authentication blockchain application stores the user ID and user wallet address at the start. This authentication step may need a few minutes to complete when Ethereum is used as a blockchain platform due to the transaction rate. The results showed that the proposed system could be very effective against some attacks like man in the middle, impersonation, replay and DoS [18].

A fog computing and blockchain-based three tier architecture is proposed that provides services for transactions and transmission near the edge in a secure manner. The proposed solution is designed for data sensitive healthcare IoT applications to provide security, reliability and authenticity. The results showed that the proposed system could effectively detect malicious nodes and is reliable. The data processing at the edge of the IoT network improves throughput and execution time [19].

For Industrial IoT (IIoT), a trusted anonymous access architecture based on a private blockchain is proposed where three different types of Software Defined Network (SDN) controllers are used for providing trusted access. To provide a balanced trade-off among credibility, confidentiality and efficiency a special module is designed in the system that shows good results in case of heavy traffic load as compared to other approaches [20].

Some of the above mentioned approaches used machine learning for device identification and fingerprinting using network traffic information or RF for feature engineering. Sensors and hubs are light weight devices and any such approach may produce a lot of overhead. Our protocol introduces minimum overhead in terms of storage and processing at sensors and hubs. Using the timing information the hub can identify each sensor individually and authenticate the data sent by that sensor.

The existing research mentioned above that used blockchain for device identification mostly requires blockchain to save device information. Our approach focuses on using blockchain to provide the validity of the sensor data stored in the database and no device information is stored on the blockchain.

Maram et al. [21] have proposed a decentralized user identity management system that provides accountability and Sybil attack resistance while being compatible with legacy web services. Users are able to recover their keys using existing online accounts using other online systems. The system has two main modules: an identity system and a key recovery system that relies on a decentralized set of nodes. Li et al. [22] proposed a blockchain-based Vehicular Digital Forensics (VDF) scheme named Eunomia to provide a secure mechanism to share data for forensic purposes with the ability to track malicious users. Even though both of the above mentioned systems provide a number of novel features, they have not been evaluated in the context of device identification.

## 3. System Overview

**Figure 1:**
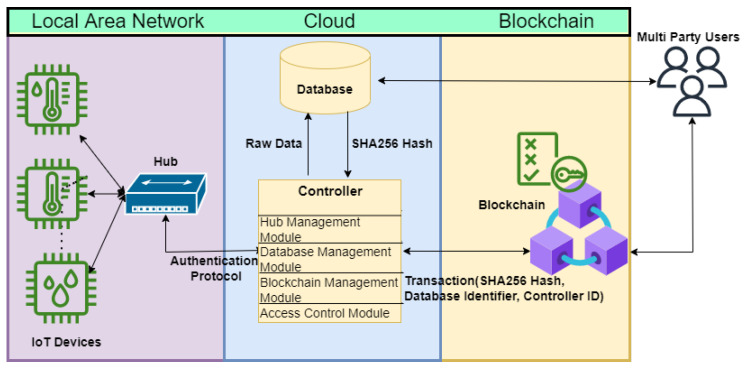
System overview.

In this section, we provide a brief overview of our system. The proposed system (Figure 1) consists of seven entities: sensors, hubs, controller, cloud, database, blockchain and users.

- Users: A user is one who can access IoT sensors, data analysis and validation services.
- IoT Device: An IoT device is a resource-constrained device that cannot run heavy cryptographic algorithms such as digital signature schemes. It is not tamper-proof, and it is connected to a device hub for networking. We assume that the connection between an IoT device and a hub is secure.
- Hubs: A hub gathers information from a group of IoT devices and sends authenticated messages to a cloud. It is a lightweight device, but it can perform cryptographic algorithms like hash functions.
- Controller: A controller collects and checks the validity of data received from hubs. It stores the validated data in an external database and updates policies (e.g., timing information) shared with the hubs. A controller can write a transaction to the blockchain periodically.
- Cloud: A cloud can have multiple controllers and a database that stores authenticated data collected from the hubs.
- Database: A database is public storage for the sensing information where a user can access their business data.
- Blockchain: A blockchain is used for auditing purposes, as a user can check the data (stored on the database) integrity by checking the transactions on the blockchain.

In our proposed system, the sensors are lightweight devices without sufficient capability for cryptographic computations. These sensors are connected to the hub and continuously sense data. These data are sent to the hub where the hub collects batches of data and sends the data to the controller periodically. A hub has limited processing and storage capability, but it is relatively stronger than IoT devices.

The controller is the main entity of the system that performs multiple functions and interacts with the hubs, database and blockchain. The controller has three main modules as discussed below:

- **Device management module:** The controller provides a timing policy to the connected hubs. The novelty of the proposed system is the timing policy which is used to identify the IoT devices. The timing policy provides the individualized data transmission schedule to all the connected hubs, which is used to identify the data sent by the valid/correct hub. The timing policy also consists of the margin of noise that every hub can add during the scheduling process.
  A controller can check the validity of the received data from the hubs. The controller uses the timing information from a group of data records coming from each hub. The controller uses the timing policy to identify the hub. Noise is a random value within a margin (decided by the controller) that is added to the scheduling interval by the hub. The controller uses an error correction technique to remove the noise correctly and identify the hub.
- **Database management module:** After identifying the source hub the SHA256 hash of the data is calculated, and the data is stored in the database. When the data is stored in the database, the index of the data is sent back to the controller.
- **Blockchain management module:** Controllers are blockchain nodes capable of generating transactions. The transaction includes SHA256 hash of the data, controller ID and the database identifier that includes database address and data index.
- **Access control module:** This module is responsible for token authentication to provide access to users.

## 4. Preliminaries

In this section, we review some technical background and give notations that will be used in this paper.

### 4.1. Secure Sketch

A secure sketch takes as input noisy information *w* and outputs a sketch s as an auxiliary string. The sketch s can be published because it does not reveal much information about *w*. Given another noisy information $w^{\prime }$, the secure sketch can reconstruct the original data *w* under the auxiliary string s, if and only if the $w^{\prime }$ is close to *w*.

**Definition** **1.** *A secure sketch consists of the following two randomized procedures (* SS,Rec *).*

- s←SS(w): on input w∈M, where M is a metric space, it outputs a sketch $s\in {\left\{0,1\right\}}^{*}$.
- *$w\leftarrow \mathrm{Rec}(w^{\prime },s)$: on input an element $w^{\prime }\in M$ and a sketch s, it outputs w if the distance dis between w and $w^{\prime }$ is not greater than a threshold t, i.e., $\mathrm{dis}(w,w^{\prime })\leq t$.*

### 4.2. Fuzzy Extractor

A fuzzy extractor extracts some randomness from noisy data *w*. Giving a similar noisy input *w*, where $w\approx w^{\prime }$, a fuzzy extractor can reproduce the same randomness. Furthermore, the generated randomness can be used. It can be derived by using a secure sketch with a strong extractor, e.g., a collision-resistant cryptographic hash function. We review a generic fuzzy extractor construction from a secure [23].

- $\left(P,R\right)\leftarrow \mathrm{Gen}\left(w;r_{1},r_{2}\right)$: on input data *w*, this algorithm generates a pair of (P,R), such that
  $$P=\left(\mathrm{SS}\left(w;r_{1}\right),r_{2}\right),\;R=\mathrm{Ext}\left(w;r_{2}\right),$$
  where SS is a secure sketch and Ext is a strong extractor.
- R←Rep: on input data $w^{\prime }$ and helper data *P*, it outputs *R*, if $\mathrm{dis}(w,w^{\prime })\leq t$.

### 4.3. $L^{p}$ Norm Based Secure Sketch

Li et al. [24] proposed a secure sketch based on Chebyshev distance. The scheme consists of three algorithms: Setup, secure sketch SS and reconstruction Rec.

- Setup: Let $L_{a}$ be a number line defined as in [24], where $L_{a}$ has exactly *v* intervals. For each interval (b,b+ka), there are ka−1 points, s.t. b+1,b+2,…,b+ka−1. *I* is an interval identifier that takes the value of the middle point of an interval. For example, I=a is an identifier of an interval (0,2a). The maximum acceptable Chebyshev distance threshold *t* is $\frac{ka}{2}$, where k=2,4,6,….
- s←SS(x): Let $x=(x_{1},x_{2},\ldots ,x_{n})$ be encoded noisy data, where $x_{i}$ is a point of $L_{a}$. This algorithm computes in three cases as follows.
  − Case 1: For all $x_{i}$, move it by $s_{i}$ to the closest interval identifier $I_{i}$, that is, $I_{i}=x_{i}+s_{i}$.
  − Case 2: If $x_{i}$ is not in any interval (e.g., the points like −ka,0,ka), it tosses a coin *c*. If c=0, it moves $x_{i}$ to the closest left interval identifier, otherwise, it moves $x_{i}$ to the right.
  − Case 3: If $x_{i}$ is the largest or the smallest point of $L_{a}$, it can be moved to either $x_{i}+\frac{ka}{2}$ or $x_{i}-\frac{ka}{2}$, depending on the toss of a coin.
  It outputs a sketch $s=(s_{1},s_{2},\ldots ,s_{n})$.
- z←Rec(y,s): on input an encoded (to $L_{a}$) data $y=(y_{1},y_{2},\ldots ,y_{n})$ and a sketch s, it runs the reconstruction procedure as follows.
  − For all $y_{i}\in y$ and $s_{i}\in s$, it calculates $y_{i}^{\prime }=y_{i}+s_{i}$.
    If $y_{i}^{\prime }>\frac{kav}{2}$, it computes $y^{\prime }=y^{\prime }-ka$.
    If $y_{i}^{\prime }<-\frac{kav}{2}$, it computes $y^{\prime }=y^{\prime }+ka$.
  − For all $y_{i}^{\prime }\in \left\{y_{1}^{\prime },y_{2}^{\prime },\ldots ,y_{n}^{\prime }\right\}$, it finds the corresponding interval identifier $I_{i}$. If $\left|I_{i}-y_{i}^{\prime }\right|>t$, this algorithm aborts and returns ⊥. Otherwise, it computes $z_{i}=I_{i}-s_{i}$. At the end, it outputs $z=(z_{1},z_{2},\ldots ,z_{n})$.

The above scheme can realize a fuzzy extractor from the generic construction (Section 4.2) by using a cryptographic hash function. In the proposed system, we use the fuzzy extractor scheme that considers time information as noisy data. We give some notations of the schemes in Table 1.

## 5. Proposed System

In this section, we present the security goals and the proposed system.

### 5.1. Security Goals

The proposed system aims to achieve secure data sharing between IoT devices, users and service providers. This work focuses on the three security aspects as follows.

- An IoT device should be identifiable without sharing secret keys. If a device is legitimate, a controller can verify the device based on its behaviors. If the device is unknown, a controller is able to recognize new devices and assign temporary access to the system. Otherwise, the system discards messages from invalid devices.
- The proposed system should provide message authentication. It is important to guarantee that the received messages are from valid IoT devices.
- The proposed system allows users to verify data integrity. The system stores IoT sensor data and provides different services for data processing. It is critical for both the cloud and users to check whether the data remains valid.

### 5.2. Lightweight Time-Based Identification Protocol (LiTBIP)

We use timing information of transactions to identify potential IoT devices. A hub collects a sequence $T=(T_{1},T_{2},\ldots ,T_{n})$ of timing information from an attached device. When receiving sensing data, the hub periodically sends it to the cloud. Meanwhile, the hub runs the proposed device identification protocol with the controller who authenticates IoT devices and checks the data integrity. Roughly speaking, a hub manipulates the time sequence and generates a secure sketch for identification. For example, the hub randomly chooses a small noisy $\Delta_{i}<\delta$, where δ is a threshold and i∈[1,n], and computes $T_{i}^{\prime }=T_{i}+\Delta_{i}$. The hub generates $T^{\prime }=\left(T_{1}^{\prime },T_{2}^{\prime },\ldots ,T_{n}^{\prime }\right)$, such that $T\approx T^{\prime }$. Then, it takes as input $T^{\prime }$ and the system parameters and runs the device identification protocol. If $\mathrm{dis}(T,T^{\prime })\leq \delta$, then the device can be identified. Otherwise, the device will be considered invalid. Note that an IoT device has a unique (sending) time sequence that may not necessarily be known to the cloud.

The proposed device identification protocol is a modified version of the work presented in [24]. The LiTBIP protocol consists of three algorithms and protocols: system setup Setup, device enrollment DeviceEnrol, and device identification DeviceIden.

**Figure 2:**
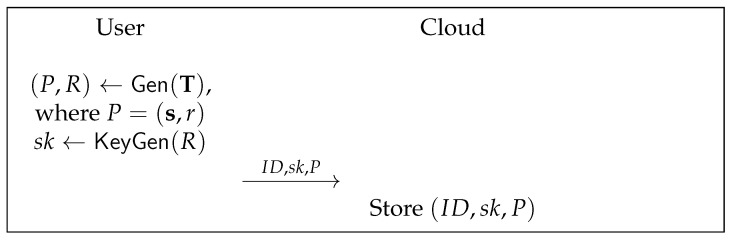
Device registration.

**Figure 3:**
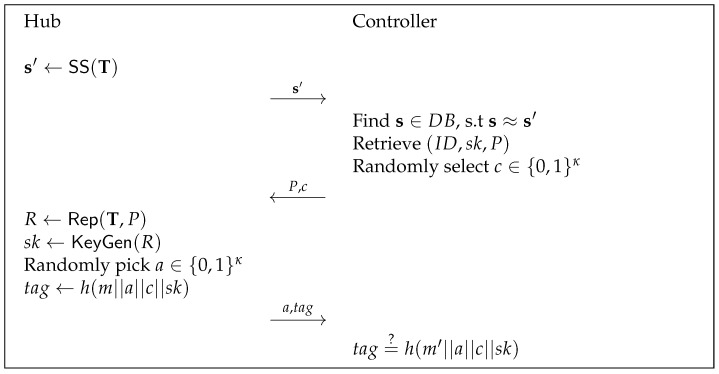
Lightweight Time-Based Identification Protocol (LiTBIP).

- Setup: The cloud server chooses a security parameter λ and a collision-resistant cryptographic hash (as a strong extractor) function $h:{\left\{0,1\right\}}^{*}\to {\left\{0,1\right\}}^{l}$. It generates a number line $L_{a}$ with the maximum acceptable Chebyshev distance *t*. Let $pp=(h,L_{a},t)$ be the public system parameters, the cloud server publishes pp.
- DeviceEnrol To register a device, a user (on behalf of of the device) interacts with the cloud. The user creates an identity ID and a time schedule T, and generates a helper data *P* and a secret key sk. The user sends (ID,sk,P) to the cloud for device registration. At the end of device registration, the cloud stores the (ID,sk,P) and allows a controller to access it. This protocol is depicted in Figure 2.
- DeviceIden To identify a device, a hub plays an interactive protocol with a controller (on behalf of a cloud). They run the protocol in the steps as follows.
  1. A hub firstly obtains the timing information when it receives the sensing data from a sensor and compiles a sequence of the time information to T. Note that the time information is considered as noisy data that contains random differences. Then, the hub performs the secure sketch algorithm SS with input T to generate a new sketch $s^{\prime }$. The hub sends $s^{\prime }$ to the controller.
  2. Upon receiving a request (i.e., $s^{\prime }$), a controller looks up the database DB and fetches a tuple (ID,sk,P), s.t. $s^{\prime }\approx s$. The controller randomly selects κ bits *c* and sends (P,c) to the hub.
  3. Upon receiving (P,c), the hub reproduces the secret key sk by using the Rep and KeyGen algorithms. It generates a κ-bit randomness and computes a message authentication code tag=h(m||a||c||sk) of a message *m*, where *m* is the sensing data of the last *n* reports.
  4. Upon receiving a response (a,tag), the controller checks if $tag=h(m^{\prime }||a||c||sk)$, where $m^{\prime }$ is the received data from the last *n* reports. If the equation holds, the hub is identified and the sensing data is authenticated.
  The identification protocol is depicted in Figure 3.

In our protocol, we assume that the time information has been encoded to the format that fits the secure sketch scheme. For example, the number line $L_{a}$ can represent a timeline where we can find time points on it. The number of time points, that is *n*, is configurable depending on the security requirements. Furthermore, the proposed protocol can identify an unknown IoT device. If a cloud allows an unknown device to be temporarily connected, for example, testing a device. The device can be automatically added to a temporary database when receiving a secure sketch from a hub. Then, the device can be identified in the following communication. However, in this mode, the device will not be authenticated because it is not registered. That is, the device does not share secret keys with the cloud. We argue that this mode facilitates the test environment.

### 5.3. Blockchain

A blockchain application is part of our proposed system to provide data integrity and can be used effectively for auditing purposes. After saving sensor data in the database, the controllers initiate transactions, which include the following data: SHA256 hash of data, controller ID and the database identifier that includes database address and data index. A smart contract will be executed to log this information into the blockchain database. Our blockchain application includes search and read functions to look for and retrieve this information from the blockchain.

### 5.4. Access Control

Data access is managed by the access control module. All users (belonging to different parties) will be allocated entitlement-based access tokens as proposed in [25]. For this multiple party access control scenario, two types of entitlement tokens (one token is for participating organizations and the other token is for individual users) will be deployed in blockchain using smart contracts. The blockchain transactions for the token deployment are digitally signed by the data owner. This token is deployed for providing access rights to other organizations and/or users to access data. Details of the entitlement right setting are outside the scope of this paper. When the users want to access data from the database, the access token and access rights will be verified by the access control module before providing users access to the data.

## 6. Implementation Details

In this section, we will provide details of our prototype implementation using Raspberry Pi and blockchain.

### 6.1. LiTBIP Implemetation Using Rasberry Pi

**Figure 4:**
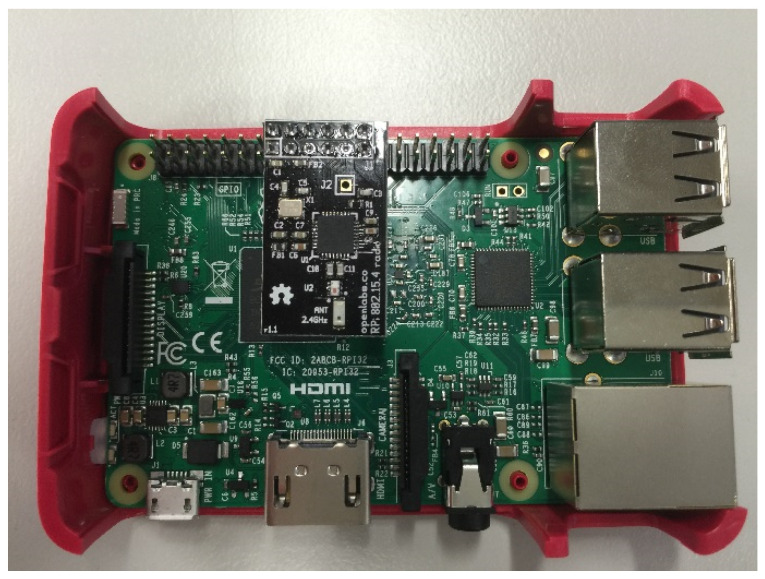
Raspberry Pi 3B+.

To evaluate the performance, we implement the proposed secure sketch scheme, fuzzy extractor and identification protocol. The implementation is conducted by using Python on Raspberry Pi 3B+ (Figure 4). The conducted performance test aims to show the (speed) performance in the different scale *d* of IoT devices. The test assumes that a time sequence has been encoded to the required format of the underlying secure sketch scheme.

Table 2 introduces the parameters used in the implementation. The number line $L_{a}$ consists of three parameters *a*, *k* and *v*. For an interval, there are at least 2 units, that is k=2. However, this setting cannot achieve constant identification in the protocol. According to the probability of false close biometric information, the value of *k* should be k∈{4,6,…}. The maximum acceptable Chebyshev distance *t* (the threshold) is set to *a* for simplicity. The implementation tested the different sizes of the input. We select a fixed dimension n=15 for T that can achieve approximately 128-bit security level.

### 6.2. Blockchain Implementation

**Figure 5:**
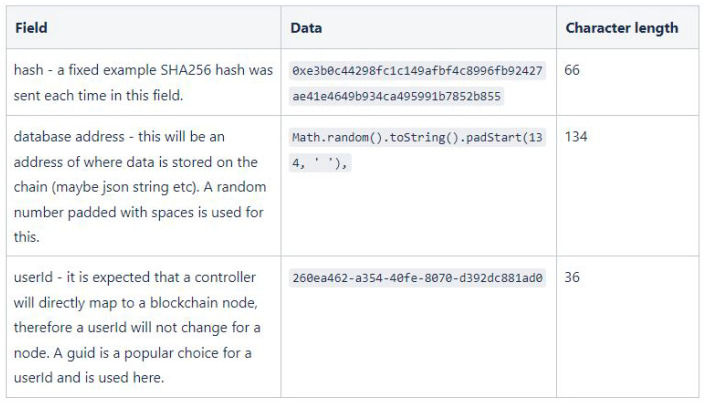
Transaction data.

The blockchain technology employed for our prototype implementation are: Geth as Ethereum client, Web3J as Java application programming interface and solidity to write the smart contract. The specifications of the machine that is used for running the prototype Ethereum blockchain are: CPU-Intel Core i5-8600K @3.6GHz, Memory-16GB ddr4 @2133MHz, Disk-Samsung SSd 970 EVO Plus 1TB, GPU-NVIDIA GeForce GTX 1080 and OS-Windows 10 Home Version 21H. As previously discussed, the transaction includes three fields as shown in Figure 5.

**Figure 6:**
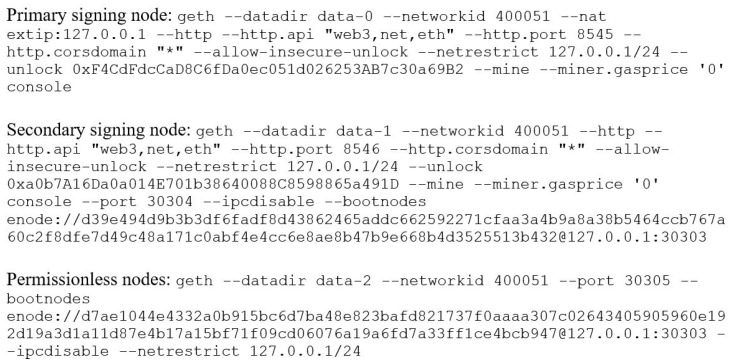
Node startup.

**Figure 7:**
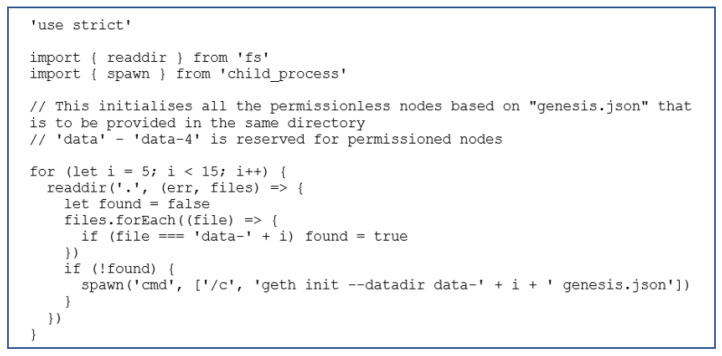
Node initialization.

These experiments are conducted with the blockchain network of up to 20 nodes where only one node is configured as a validating node to run the Proof-of-Authority (PoA) consensus algorithm. The network consists of a varying number of controller nodes that are sending the transactions to the validating node. Figure 6 shows the configuration of validating and controller nodes. Each node requires setup and initialization, and configuring this on a large scale would require repetitive manual work. Therefore, a script was created to initialize and run each node via the Geth CLI. A snippet of the script is shown in Figure 7. This script was written in Node.js; the details of how it works are available in the script’s comments. Figure 7 shows the script that initializes all the permissionless nodes based on “genesis.json” that is to be provided in the same directory.

This is broken into two scripts, one to handle the initialization of all nodes based on the genesis file (run this first) and one to run all of the nodes and exit them gracefully. Currently, these scripts only handle the creation and startup of nodes without signing authority. These scripts are designed to run on Windows Operating System only.

**Figure 8:**
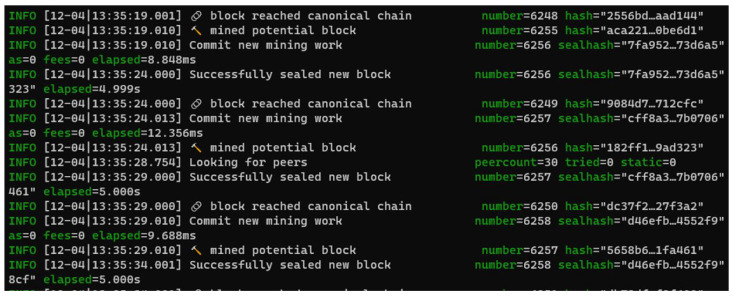
Validating node with 5 s block period.

**Figure 9:**
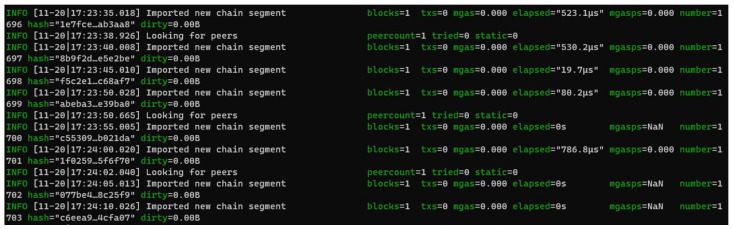
Node syncing.

**Figure 10:**
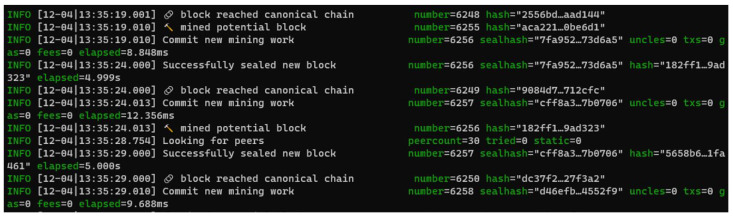
Node 0 syncing with 30 peers.

Figure 8 shows successful mining of a block in every 5 s period. Figure 9 shows another node (permissionless) is syncing with the signing node. We can see that this node is importing the latest chain segment each time it arrives. It has one peer—the first node, which is set up as the signing node. Figure 10 shows that node0, which is the original node that mined the first block, is syncing with 30 peers. It is shown that this mode is mining new blocks—the peers will import these added chain segments.

**Figure 11:**
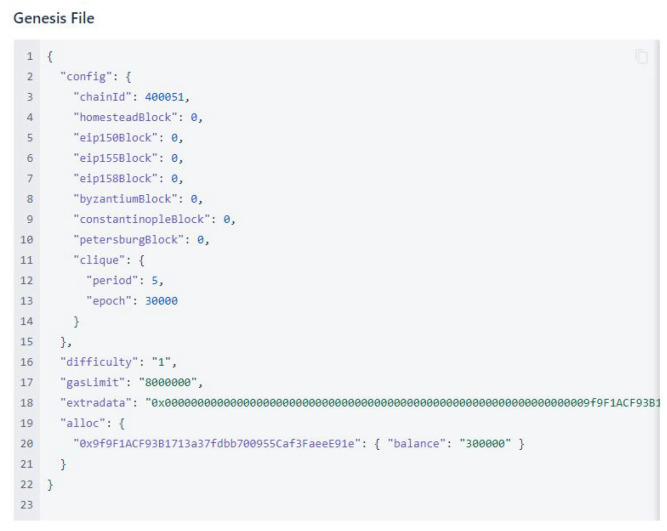
Genesis file.

For the PoA consensus algorithm, the genesis block was configured with a different block period; one test configuration is shown in Figure 11.

**Figure 12:**
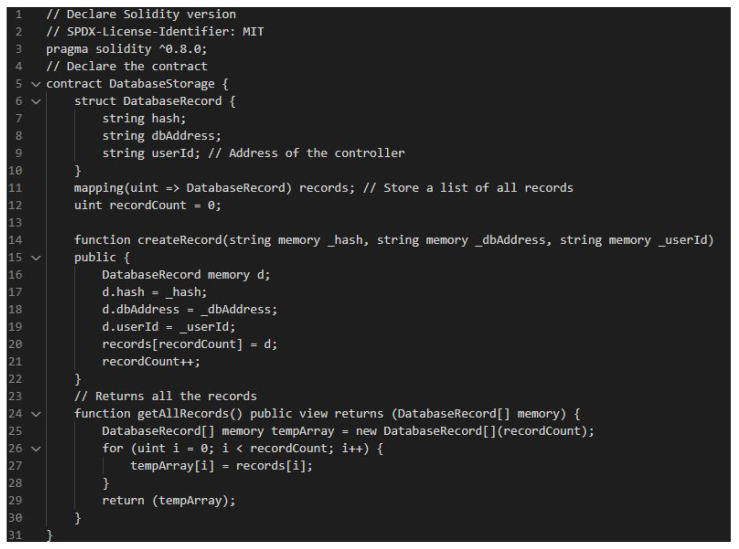
Smart contract.

**Figure 13:**
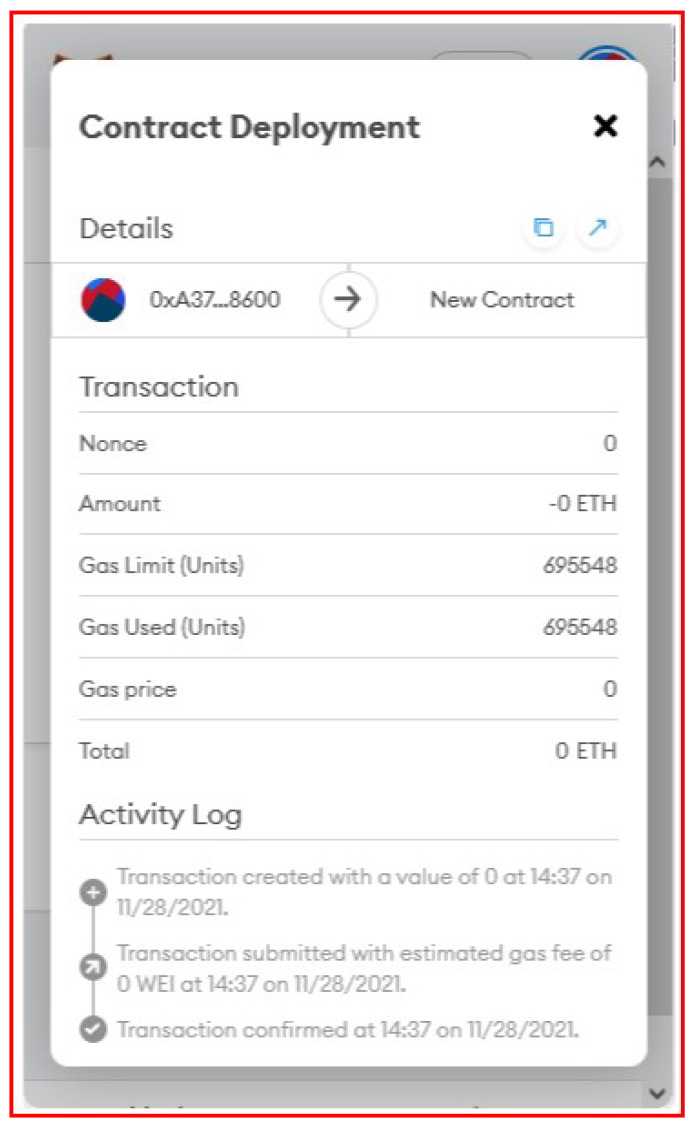
Smart contract deployment in blockchain.

Our smart contract is written using solidity language and it consists of two functions such as *createRecord()* and *getAllRecords()* as shown in Figure 12. The data structure used to store the data values is shown in lines 6-10 in the figure. *createRecord()* function is used for committing the transactions and *getAllRecords()* is used to retrieve information from the blockchain. The smart contract is deployed in our permissioned Ethereum network and used for storing and retrieving data to provide data integrity in our proposed system. Figure 13 shows the deployment of smart contract in blockchain.

We have done thorough system testing to confirm that the blockchain nodes have been created successfully and blockchain transactions could be logged successfully. The initial testing was completed using one “permissioned” node to sign transactions (ran manually from a PowerShell terminal) and 20 “permissionless” nodes (ran via the Node.js script). Using an automated Node.js script, the 20 permissionless nodes continuously sent transactions, and exactly 50 transactions were able to be committed per block. One account per blockchain node was used. This was done to represent the 1-to-1 mapping of a blockchain node and controller. The number of transactions that are mined per block is dependent on the size of the data (at least for data this small), as the same setup was tested with the string “a” in all the fields and 85 transactions per block were observed. Signing and sending the transactions was the most performance-intensive part.

## 7. Performance Evaluation

In this section, we discuss the performance evaluation of our preliminary prototype implementation.

### 7.1. LiTBIP Evaluation

In our experiment, we evaluate the computational overhead of the identification protocol. The performance of our device identification protocol is nearly constant, even when a million hubs were included in the experiments. This is consistent with the earlier work’s [24] secure sketch protocol, which takes around 40 milliseconds in a large-scale setting. The major overhead of different scales is the time taken to look up the data records, which is still negligible. This demonstrates the efficiency and feasibility of the proposed system in practice.

### 7.2. Blockchain Performance Evaluation

**Figure 14:**
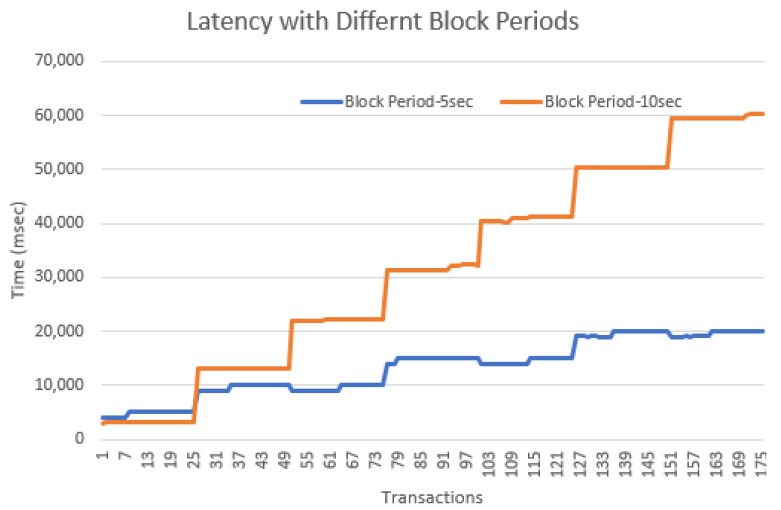
Latency with different block periods.

We have used different types of configurations and analyzed the performance of our prototype implementation. For blockchain applications, one of the major performance concerns is the time taken to complete a transaction (latency) [26]. The time latency of a transaction is calculated as the time from when a controller submits a transaction to the creation of the block that contains that transaction. Figure 14 shows transaction latency when the mining times are 5 s and 10 s with ten controller nodes and one “permissioned” node. It is apparent from the results that latency depends on the block period; hence, depending upon the system configuration the block period should be adjusted. One important factor is the frequency of transactions by the controllers that depends on hub and controller communication: for example, the controllers send a few transactions when the hub data of a long period (12 h or 24 h) is authenticated in one transaction. We have evaluated our prototype implementation with a few other settings by varying mining intervals (the time to mine a block) and the block size. The general trend of the results is the same, performance has an inverse relationship with the block period. Our preliminary results demonstrate that the proposed system is easily implementable. However, the scalability of the application depends on the mining interval. We are currently working on developing a new approach to making the system more scalable.

## 8. Conclusions

In this paper, we proposed a novel lightweight fuzzy extractor and blockchain based secured IoT system that provides data security in two ways. Our system consists of a novel Light weight Time-based Identification Protocol for small IoT devices. Data security is also provided by employing blockchain application in the system for easy data sharing and auditing.

We also presented our implementation work of LiTBIP on the Raspberry Pi platform to evaluate the effectiveness of our protocol for a large number of hubs. The scalability analysis of the protocol showed that the computation overhead is very small and constant which makes it an excellent choice for large scale IoT systems. We have developed and evaluated a prototype blockchain implementation that captures the main functionality of the proposed system at the blockchain end. The performance evaluation showed that a significant number of transactions could be handled by our proposed blockchain application.

This paper provides the details of the preliminary evaluation of our proposed approach, we are planning to deploy our system in a real-world environment with a large number of IoT devices. Such large scale real world deployment may introduce new challenges, such as data integration and configuration issues, that we will need to look into.

## Figures and Tables

**Table 1:** Notations of the schemes.

Notations	Description
T:	time information, a vector a points on $L_{a}$.
DB:	a database stores device information, shared secret keys and helper data.
$s\;\approx \;s^{\prime }$:	vectors s and $s^{\prime }$ are close under some measurement.
sk:	a secret key shared between a hub and a cloud.
${\left\{0,1\right\}}^{\kappa }$:	a κ-bit value.
KeyGen:	a key generation algorithm of secret keys.
Gen:	a generation procedure of a fuzzy extractor.
Rep:	a reproduction procedure of a fuzzy extractor.
dis(x,y):	a function returns distance between x and y.

**Table 2:** Implementation parameters of our protocol.

Parameter	Value
*a*	100
*k*	4
*v*	500
*n*	15
*t*	100
κ	128
*d*	[100, 1,000,000]
Random Extractor	SHA256
